# Surgical and oncological outcomes of robotic- versus laparoscopic-assisted distal gastrectomy with D2 lymphadenectomy for advanced gastric cancer: a propensity score‑matched analysis of 1164 patients

**DOI:** 10.1186/s12957-022-02778-w

**Published:** 2022-09-28

**Authors:** Gengmei Gao, Hualin Liao, Qunguang Jiang, Dongning Liu, Taiyuan Li

**Affiliations:** 1 The Department of Gastrointestinal Surgery, the First Affiliated Hospital of Nanchang University, Jiangxi Province, Nanchang, 330006 China; 2 Department of Graduate School, Medical College of Nanchang University, Jiangxi Province, Nanchang, 330006 China; 3 Department of General Surgery, The First Affiliated Hospital of Nanchang University, Nanchang, 330000 China

**Keywords:** Advanced gastric cancer, Robotic distal gastrectomy, Laparoscopic distal gastrectomy, Oncologic outcome

## Abstract

**Background:**

Studies on surgical outcomes after robotic surgery are increasing; however, long-term oncological results of studies comparing robotic-assisted distal gastrectomy (RADG) versus laparoscopic-assisted distal gastrectomy (LADG) for advanced gastric cancer (AGC) are still limited. This study aimed to assess the surgical and oncological outcomes of RADG and LADG for the treatment of AGC.

**Methods:**

A total of 1164 consecutive AGC patients undergoing RADG or LADG were enrolled between January 2015 and October 2021. Propensity score-matched (PSM) analysis was performed to minimize selection bias. The perioperative and oncological outcomes between the two groups were compared.

**Results:**

Patient’s characteristics were comparable between the two groups after PSM. RADG group represented a longer operative time (205.2 ± 43.1 vs 185.3 ± 42.8 min, *P* < 0.001), less operative blood loss (139.3 ± 97.8 vs 167.3 ± 134.2 ml, *P* < 0.001), greater retrieved lymph nodes (LNs) number (31.4 ± 12.1 vs 29.4 ± 12.3, *P* = 0.015), more retrieved LNs in the supra-pancreatic areas (13.4 ± 5.0 vs 11.4 ± 5.1, *P* < 0.001), and higher medical costs (13,608 ± 4326 vs 10,925 ± US $3925, *P* < 0.001) than LADG group. The overall complication rate was 13.7% in the RADG group and 16.6% in the LADG group, respectively; the difference was not significantly different (*P* = 0.242). In the subgroup analysis, the benefits of RADG were more evident in high BMI patients. Moreover, the 3-year overall survival (75.5% vs 73.1%, *P* = 0.471) and 3-year disease-free survival (72.9% vs 71.4%, *P* = 0.763) were similar between the two groups.

**Conclusion:**

RADG appears to be a safe and feasible procedure and could serve as an alternative treatment for AGC in experienced centers.

## Introduction

Gastric cancer (GC) has been a significant public health concern worldwide due to its high morbidity and mortality, especially in East Asia [1, 2]. In China, AGC patients account for more than 80% of the total GC patients. Moreover, radical gastrectomy with LN dissection remains the mainstream treatment for AGC patients [2, 3]. In contrast, with better perioperative outcomes and comparable oncological outcomes, minimally invasive treatments for AGC have been embraced over open gastrectomy [4, 5]. LADG was first reported by Kitano et al. in 1994 [6]. Since then, numerous studies have been carried out to compare laparoscopic gastrectomy (LG) and open gastrectomy for GC. The results confirmed that compared with open gastrectomy, LG reduced blood loss, postoperative complications, and postoperative hospital stay with equivalent oncological outcomes [7–11]. However, laparoscopy-induced technical limitations and drawbacks, such as limited movement of laparoscopic instruments, long learning curve, and a decreased tactile sensation, hindered the performance of LN dissection [10, 12, 13]. Furthermore, for AGC patients, high body mass index (BMI) may further damage the quality of D2 lymphadenectomy [14].

Additionally, a robotic surgical system has been introduced to address the limitations of laparoscopy and is welcomed in GC treatment owing to its satisfying early postoperative outcomes [13, 15–17]. Robotic gastrectomy (RG) has been confirmed to be a safe and feasible technique with better anatomical and operative conditions and comparable long-term results compared to LG [12, 15, 18–20]. However, most studies were limited to short-term outcomes, unmatched groups, small sample sizes, and early GC. On this basis, we conducted the study with a sample size including 820 patients for comparison of the surgical and oncological outcomes of RADG and LADG for AGC using a PSM analysis.

## Materials and methods

### Patients

**Fig. 1:**
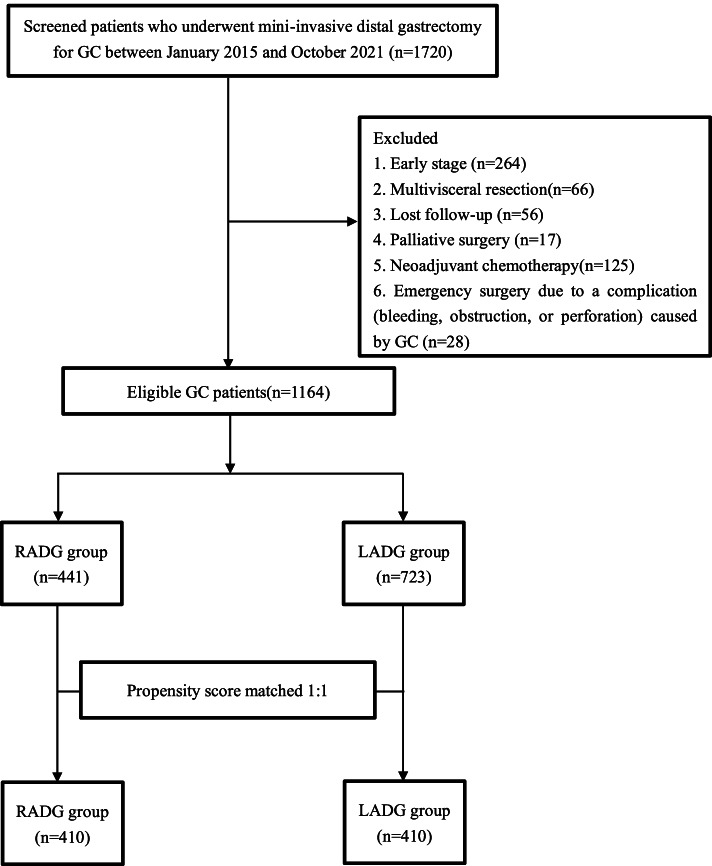
Flow chart of patient selection

RG was first performed to the Department of Gastrointestinal Surgery at the First Affiliated Hospital of Nanchang University in January 2015. To assess surgical and oncological outcomes, we retrospectively reviewed a clinical database of GC patients to identify 1720 patients undergoing RADG or LADG between January 2015 and October 2021. The study design was presented in Fig. 1. Ethical approval for this study was received from the Research Ethics Committee of the First Affiliated Hospital of Nanchang University. All patients and their family members provided written, informed consent for their operations after receiving a comprehensive and detailed explanation of the surgical and oncological risks. In addition, all patients received preoperative staging based on the preoperative examination, such as gastroscopy, biopsy, and chest and abdominopelvic enhanced computed tomography. The exclusion criteria were as follows: (1) neoadjuvant therapy or radiation therapy; (2) depth of invasion confined to pTis, pT1, or pT4b; (3) multivisceral resection; (4) lost follow-up; (5) incomplete clinical records; (6) palliative surgery; and (7) emergency surgery. Finally, a total of 1164 patients were included in this analysis (441 in RADG group and 723 in LADG group). In order to minimize the impact of potential bias due to imbalanced clinicopathological parameters in the current study, PSM analysis was conducted through a logistic regression model which included the following variables: age, tumor size, previous abdominal surgery, sex, pathologic T stage, type of reconstruction, pathologic N stage, histology type, BMI, and American Society of Anesthesiologists (ASA) score. RADG and LADG groups were matched with a caliper width of 0.02 [21, 22]. Finally, there were 410 patients in each group after PSM. The patient staging was evaluated based on the American Joint Committee on Cancer Staging (the eighth edition) [23]. Postoperative complications were examined according to the Clavien-Dindo classification system [24]. Patients were categorized into the normal *(BMI* ≤ 24 kg/m^2^) or the high BMI group (*BMI* > 24 kg/m^2^) based on BMI values [25].

### Surgical procedure

All AGC patients undergoing radical distal gastrectomy with D2 lymphadenectomy based on the Japanese Gastric Cancer Treatment Guidelines received three experienced surgeons as we previously described [13, 26, 27]. Surgical methods (RADG and LADG) were chosen in accordance with the wishes of patients and their family members after they were informed of the potential technical superiority and disadvantages of RADG and LADG. The details of the LN dissections and gastrectomy during the RATG procedures did not markedly differ from those during the LATG procedures, except for using articulating robotic instruments. After lymphadenectomy, a 5–7 cm incision was used for digestive tract anastomosis and specimen removal. It should be emphasized, however, that the indocyanine green navigation technique was not used in either group. The reconstruction method was determined based on the experience of surgeons and tumor location. Surgical procedures for RADG and LADG were previously detailed [27].

### Follow‑up

Patients were followed up every 3 months for the first year after surgery and then every 6 months for the next 4 years. In addition, blood routine examinations including serum carcinoembryonic, gastrointestinal endoscopy, and abdominopelvic computed tomography were performed for regular follow-up. Survival time was determined as the time from the operation date to the date of a new event or last follow-up. DFS was calculated from the surgery date to the date of recurrence or death. Cancer recurrence was evaluated using chest and abdominopelvic enhanced computed tomography. According to the postoperative pathological stage, the adjuvant 5-fluorouracil (5-FU) chemotherapy was routinely administered to patients with tumors of pathologic stages 2 and 3.

### Statistical analysis

Statistical analyses and PSM were performed in the present study using SPSS 26.0 software (IBM Corporation, Armonk, NY, USA). Continuous variables were presented as the mean ± standard deviation and compared between groups using the Mann–Whitney *U*-test or Student’s *t*-test. Qualitative variables were described as the number (%), and the chi-square test or Fisher’s exact test was performed for qualitative variables. Survival curves were plotted using the Kaplan–Meier method. The log-rank test was used for the comparison of survival curves. The independent risk factors for overall survival (OS) and disease-free survival (DFS) were identified based on the cox proportional-hazards regression model. Variables with *P*-values less than 0.1 in the univariate analysis were included in the multivariate analysis. *P* < 0.05 was considered statistically significant, and all *P*-values were two sided.

## Results

### Patient characteristics

The clinicopathological characteristics of patients were detailed in Table 1. A total of 1164 AGC patients were collected in the present study, of whom 441 received RADG and 723 underwent LADG. In the entire cohort, LADG group included more patients with previous abdominal surgery (5.7% vs. 16.9%, *P* < 0.001). Besides, significant differences were found between the two groups in the reconstruction type, histology type, and pathologic T stage (*P* < 0.05). The general background clinical variables, including age, sex, pathologic T stage, type of reconstruction, pathologic N stage, tumor size, BMI, ASA score, and pathologic TNM stage, had no significant difference between the two groups. Following PSM, there were 410 patients in each group, and all clinicopathological parameters were well-balanced between two groups (Table 1).

**Table 1:** Clinicopathological characteristics of patients in the entire and PSM cohorts

Variables	Entire cohort		*P*	PSM cohort		*P*
	RADG (*n* = 441)	LADG (*n* = 723)		RADG (*n* = 410)	LADG (*n* = 410)	
Age, years	59.9 ± 11.5	59.7 ± 11.4	0.826	59.8 ± 10.8	59.7 ± 11.0	0.839
Sex, *n* (%)			0.768			0.189
Male	308 (69.8)	499 (69.0)		284 (69.3)	301 (73.4)	
Female	133 (30.2)	224 (31.0)		126 (30.7)	109 (26.7)	
BMI, kg/m^2^	23.2 ± 3.2	23.1 ± 3.1	0.472	23.1 ± 3.1	23.1 ± 3.0	0.971
ASA score, *n* (%)			0.630			0.741
1	211 (47.8)	325 (45.0)		203 (49.5)	198 (48.3)	
2	154 (34.9)	267 (36.9)		133 (32.4)	143 (34.9)	
3	76 (17.3)	131 (18.1)		74 (18.1)	69 (16.8)	
Tumor size, cm	4.1 ± 2.1	4.3 ± 2.6	0.233	4.1 ± 2.1	4.2 ± 2.2	0.642
Previous abdominal surgery, *n* (%)			< 0.001			0.544
No	416 (94.3)	601 (83.1)		389 (94.9)	385 (94.0)	
Yes	25 (5.7)	122 (16.9)		21 (5.1)	25 (6.0)	
Type of reconstruction, *n* (%)			< 0.001			0.939
B-I	142 (32.2)	370 (51.2)		134 (32.7)	138 (33.7)	
B-II	258 (58.5)	260 (36.0)		244 (59.5)	239 (58.3)	
Roux-en-Y	41 (9.3)	93 (12.8)		32 (7.8)	33 (8.0)	
Histology type, *n* (%)			0.019			0.806
Well/moderately	101 (22.9)	211 (29.2)		96 (23.4)	99 (24.1)	
Poorly/undifferentiated	340 (77.1)	512 (70.8)		314 (76.6)	311 (75.9)	
Pathologic T stage, *n* (%)			< 0.001			0.879
T2	193 (43.8)	177 (24.5)		172 (42.0)	165 (40.2)	
T3	138 (31.3)	245 (33.9)		132 (32.2)	137 (33.4)	
T4a	110 (24.9)	301 (41.6)		106 (25.8)	108 (26.4)	
Pathologic N stage, *n* (%)			0.732			0.942
N0	190 (43.1)	323 (44.7)		182 (44.4)	188 (45.9)	
N1	73 (16.6)	113 (15.6)		70 (17.1)	68 (16.6)	
N2	82 (18.6)	119 (16.5)		80 (19.5)	82 (20.0)	
N3	96 (21.7)	168 (23.2)		78 (19.0)	72 (17.5)	
Pathologic TNM stage, *n* (%)			0.079			0.970
IB	50 (11.3)	99 (14.0)		48 (11.7)	52 (12.7)	
IIA	36 (8.2)	76 (10.5)		34 (8.3)	38 (9.3)	
IIB	83 (18.8)	110 (15.1)		73 (17.8)	71 (17.3)	
IIIA	64 (14.5)	130 (17.9)		60 (14.6)	58 (14.1)	
IIIB	88 (20.0)	148 (20.4)		87 (21.2)	79 (19.3)	
IIIC	120 (27.2)	160 (22.1)		108 (26.4)	112 (27.3)	

*RADG* robotic-assisted distal gastrectomy, *LADG* laparoscopic-assisted distal gastrectomy, *PSM* propensity score matching, *BMI* body mass index, *ASA* American Society of Anesthesiologists, *TNM* tumor-node-metastasis

### Surgical outcomes of cohorts

The surgical outcomes were displayed in Table 2. In the PSM cohort, RADG group represented a longer operative time (205.2 ± 43.1 vs 185.3 ± 42.8 min, *P* < 0.001), less operative blood loss (139.3 ± 97.8 vs 167.3 ± 134.2 ml, *P* < 0.001), greater retrieved LN number (31.4 ± 12.1 vs 29.4 ± 12.3, *P* = 0.015), more retrieved LNs in the supra-pancreatic areas (13.4 ± 5.0 vs 11.4 ± 5.1, *P* < 0.001), and higher medical costs (13,608 ± 4326 vs 10,925 ± US $3925, *P* < 0.001) than LADG group. Moreover, compared with the LADG group, the RADG group showed comparable outcomes with regard to time to first liquid diet (3.3 ± 1.3 vs 3.3 ± 1.6 days, *P* = 0.566), length of postoperative hospital stay (9.0 ± 3.9 vs 9.1 ± 3.5 days, *P* = 0.371), and time to first flatus (2.8 ± 1.3 vs 2.7 ± 1.4 days, *P* = 0.219). Regarding the entire cohort, results remained almost identical (Table 2).

**Table 2:** Comparison of surgical outcomes and postoperative recovery

Variables	Entire cohort		*P*	PSM cohort		*P*
	RADG (*n* = 441)	LADG (*n* = 723)		RADG (*n* = 410)	LADG (*n* = 410)	
Operation time, min	206.0 ± 43.6	189.4 ± 46.3	< 0.001	205.2 ± 43.1	185.3 ± 42.8	< 0.001
Operative blood loss, mL	138.8 ± 98.6	165.6 ± 135.3	< 0.001	139.3 ± 97.8	167.3 ± 134.2	< 0.001
Conversion, *n* (%)	3 (0.6)	10 (1.4)	0.268	3 (0.7)	6 (1.5)	0.315
Time to first flatus, days	2.9 ± 1.4	2.7 ± 1.5	0.157	2.8 ± 1.3	2.7 ± 1.4	0.219
Time to first liquid diet, days	3.3 ± 1.4	3.5 ± 1.8	0.632	3.3 ± 1.3	3.3 ± 1.6	0.566
Postoperative hospital stay, day	8.8 ± 4.3	9.3 ± 5.1	0.066	9.0 ± 3.9	9.1 ± 3.5	0.371
Retrieved LN number	31.6 ± 12.1	29.8 ± 12.0	0.013	31.4 ± 12.1	29.4 ± 12.3	0.015
Retrieved supra- pancreatic LN	13.5 ± 5.2	11.6 ± 5.0	< 0.001	13.4 ± 5.0	11.4 ± 5.1	< 0.001
Medical cost, dollars	13,346 ± 4356	10,815 ± 4325	< 0.001	13,608 ± 4326	10,925 ± 3925	< 0.001

*RADG* robotic-assisted distal gastrectomy, *LADG* laparoscopic-assisted distal gastrectomy, *PSM* propensity score matching, *LN* lymph node

The postoperative complications and mortality were displayed in Table 3. The overall complication rate showed no difference between the two groups in the entire and PSM cohorts (15.2% vs. 18.3%, *P* = 0.178; 13.7% vs. 16.6%, *P* = 0.242, respectively). In the entire and PSM cohorts, we found that the grade 2 complications were more common in the two groups. Besides, the incidence of severe complications (Clavien-Dindo grade > II) between the two groups did not differ in the entire and PSM cohorts (5.2% vs 8.2%, *P* = 0.057; 4.9% vs 6.3%, *P* = 0.363, respectively). The postoperative mortality presented no difference between the two groups in the entire and PSM cohorts (0.6% vs 1.2%, *P* = 0.355; 0.6% vs 1.0%, *P* = 0.704, respectively).

**Table 3:** Postoperative morbidity and mortality

Variables	Entire cohort		*P*	PSM cohort		*P*
	RADG (*n* = 441)	LADG (*n* = 723)		RADG (*n* = 410)	LADG (*n* = 410)	
Overall complications, *n* (%)	67 (15.2)	132 (18.3)	0.178	56 (13.7)	68 (16.6)	0.242
Clavien-Dindo grade						
I	24 (5.4)	35 (4.8)	0.650	18 (4.4)	20 (4.9)	0.740
Wound problem	8	10		7	8	
Fever	4	9		4	3	
Cardiac dysfunction	7	10		4	7	
Diarrhea	5	6		3	2	
II	20 (4.5)	38 (5.3)	0.584	17 (4.1)	22 (5.4)	0.412
Wound infection	3	6		2	3	
Intra-abdominal infection	4	5		4	3	
Intestinal obstruction	6	5		5	3	
Pulmonary infection	5	8		5	6	
Pleural effusion	1	5		0	3	
Anastomotic leakage	1	4		1	2	
Intra-abdominal bleeding	0	3		0	1	
Duodenal stump leakage	0	2		0	1	
III	12 (2.7)	30 (4.1)	0.205	11 (2.7)	15 (3.7)	0.425
Wound problem	2	4		2	2	
Duodenal stump leakage	4	5		3	2	
Anastomotic leakage	3	7		3	4	
Pancreatic fistula	2	6		2	3	
Intra-abdominal infection	1	8		1	4	
IV	8 (1.8)	20 (2.8)	0.304	7 (1.7)	7 (1.7)	1.000
Respiratory failure	5	12		4	5	
Cardiac failure	3	8		3	2	
V	3 (0.8)	9 (1.3)	0.355	3 (0.7)	4 (0.9)	0.704
Clavien-Dindo grade > II	23 (5.2)	59 (8.2)	0.057	20 (4.9)	26 (6.3)	0.363
Postoperative mortality, *n* (%)	3 (0.6)	9 (1.2)	0.355	3 (0.6)	4 (1.0)	0.704

*RADG* robotic-assisted distal gastrectomy, *LADG* laparoscopic-assisted distal gastrectomy, *PSM* propensity score matching

### Subgroup comparison according to BMI

We investigated AGC patients by grouping them according to BMI. The perioperative outcomes of the subgroup comparison were given in Table 4. As can be seen, operative blood loss, retrieved LN number, and retrieved LNs in the supra-pancreatic areas were significantly different between RADG and LADG groups in both high and normal BMI subgroups (*P* < 0.001). Furthermore, subgroup analysis of BMI indicated that RADG group showed a longer operation time than LADG group in normal BMI patients, while no significant difference existed between the two groups in high BMI patients. Additionally, in the subgroup of high BMI patients, the incidence of severe complications in RADG group was significantly lower than that in LADG group (1.2% vs 3.4%, *P* = 0.037).

**Table 4:** Subgroup comparison of the two surgery methods in different body mass index

Variables	BMI > 24		*P*	BMI ≤ 24		*P*
	RADG (*n* = 111)	LADG (*n* = 105)		RADG (*n* = 299)	LADG (*n* = 305)	
Age, years	60.2 ± 10.5	60.4 ± 12.0	0.857	60.7 ± 11.1	60.4 ± 11.7	0.718
Gender, *n* (%)			0.564			0.059
Male	76 (68.5)	68 (64.8)		208 (70.0)	233 (77.9)	
Female	35 (31.5)	37 (35.2)		91 (30.0)	72 (32.1)	
BMI, kg/m^2^	27.0 ± 1.9	26.8 ± 2.1	0.292	21.5 ± 1.9	21.7 ± 2.0	0.172
Operative time, min	214.8 ± 41.8	209.3 ± 42.9	0.336	196.5 ± 35.4	163.2 ± 36.5	0.000
Operative blood loss, mL	142.2 ± 96.5	178 ± 102.6	< 0.001	131.5 ± 90.2	158.6 ± 106.8	< 0.001
Conversion, *n* (%)	1 (0.2)	5 (1.2)	0.084	2 (0.4)	1 (0.2)	0.551
Time to first flatus, days	2.8 ± 1.2	2.7 ± 1.1	0.261	2.8 ± 1.3	2.7 ± 1.4	0.219
Time to first liquid diet, days	3.1 ± 1.2	3.4 ± 1.3	0.121	3.3 ± 1.3	3.0 ± 1.4	0.089
Postoperative hospital stay, day	9.2 ± 3.8	9.3 ± 3.4	0.356	8.8 ± 3.6	9.2 ± 3.6	0.378
Retrieved LN number	30.8 ± 12.5	27.1 ± 12.3	0.007	33.6 ± 13.1	31.8 ± 12.6	0.013
Retrieved supra-pancreatic LN	13.3 ± 3.9	11.2 ± 4.8	0.001	13.5 ± 5.2	11.8 ± 5.6	0.002
Overall complications, *n* (%)	18 (4.4)	26 (6.3)	0.215	38 (9.3)	42 (10.2)	0.638
Clavien-Dindo grade > II	5 (1.2)	14 (3.4)	0.037	15 (3.7)	12 (2.9)	0.557
Postoperative mortality, *n* (%)	1 (0.2)	2 (0.5)	0.563	2 (0.5)	2 (0.5)	1.000

*RADG* robotic-assisted distal gastrectomy, *LADG* laparoscopic-assisted distal gastrectomy, *LN* lymph node, *BMI* body mass index

### Long‑term oncologic outcomes for PSM cohort

**Fig. 2:**
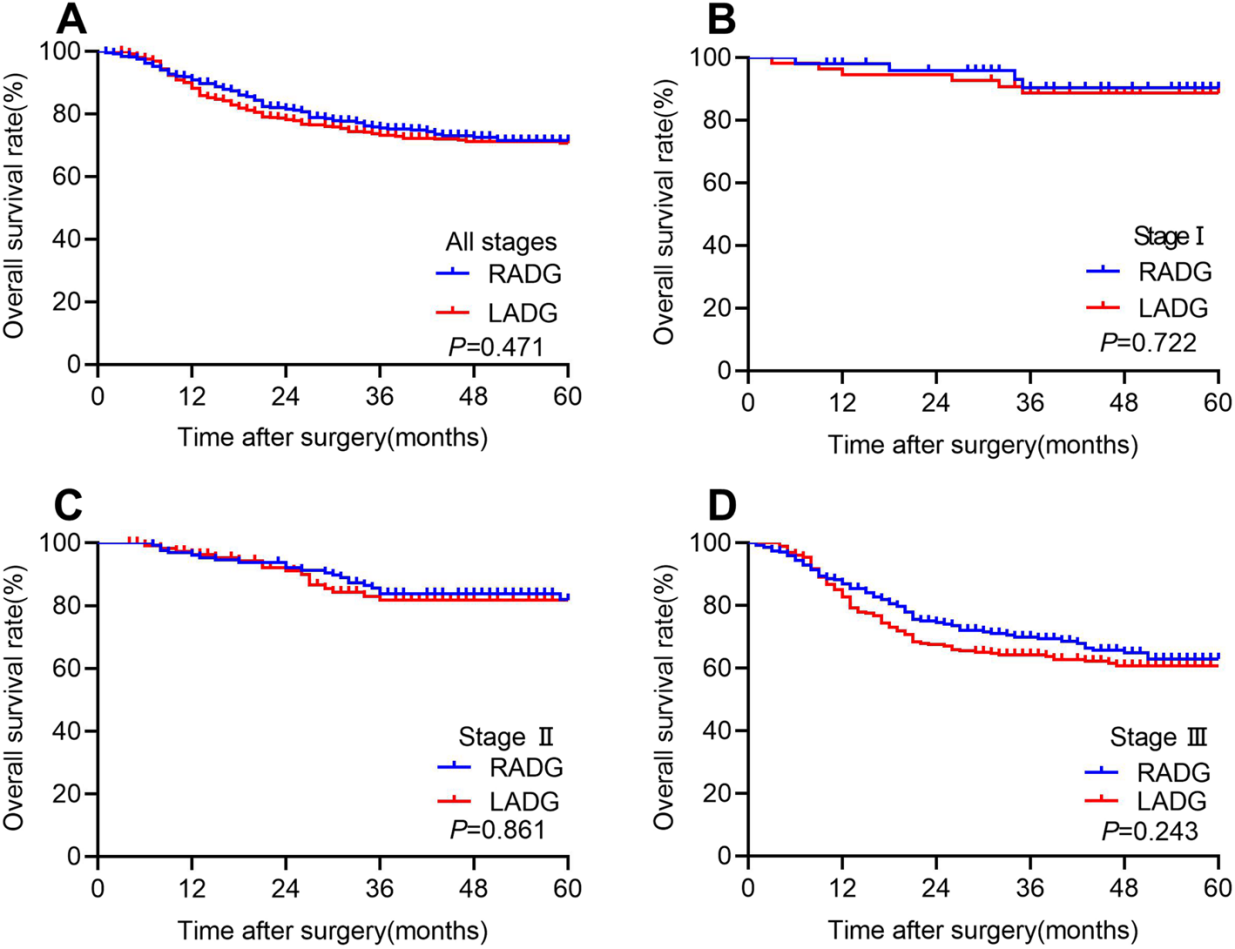
Kaplan–Meier estimates of overall survival of patients with all stages (**A**), patients with stage 1 (**B**), patients with stage 2 (**C**), and patients with stage 3 (**D**)

**Fig. 3:**
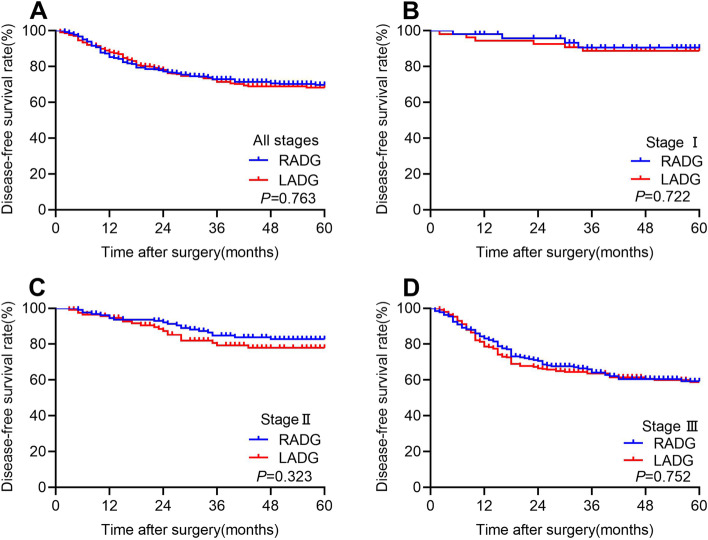
Kaplan–Meier estimates of disease-free survival of patients with all stages (**A**), patients with stage 1 (**B**), patients with stage 2 (**C**), and patients with stage 3 (**D**)

The follow-up endpoint was February 2022. The median follow-up period was 39 months (range: 4–84). The 3-year OS rate was comparable between RADG and LADG groups (75.5% vs. 73.1% *P* = 0.471; Fig. 2A). The 3-year OS rate between the two groups at each stage showed no significant difference (stage 1B 90.3% vs. 88.6, *P* = 0.722; stage 2 83.8% vs. 81.7, *P* = 0.861; stage 3 69.4% vs. 63.9, *P* = 0.243; Fig. 2 B–D). During the follow-up period, 115 (28.0%) patients experienced recurrence or death in RADG group, compared to 126 (30.7%) patients in LADG group. The 3-year DFS rate showed no significant difference between RADG and LADG groups (72.9% vs. 71.4%, *P* = 0.763; Fig. 3A). The 3-year DFS rates were still comparable between the two groups in each stage respectively (stage 1B 90.5% vs. 88.8%, *P* = 0.722; stage 2 84.7% vs. 79.3%, *P* = 0.323; stage 3 64.1% vs. 63.6%, *P* = 0.752; Fig. 3 B–D).

Cox proportional-hazards regression model was conducted to identify risk factors associated with OS and DFS following RADG and LADG for AGC in the PSM cohort. A low OS was associated with patients with the following conditions: age ≥ 65, *BMI* < 25 kg/m^2^, tumor size ≥ 4 cm, and stage T3-T4a, stage N1-N3, stages 2A–3C in univariate analysis (all *P*-values < 0.1, Table 5). Furthermore, multivariate analyses identified the stage T3-T4a, stage N1-N3, and stages 2A–3C as independent risk factors for OS. However, the robotic surgery approach failed to be an independent risk factor for OS (*HR* 0.918, 95% *CI* 0.707–1.192, *P* = 0.521). Regarding DFS, results similar to those for OS were obtained (Table 6).

**Table 5:** Univariate and multivariate analyses of the risk factors for overall survival in the PSM cohort

Variables	Univariate		*P*	Multivariate		*P*
	HR	95% *CI*		HR	95% *CI*	
Age, years			0.036			0.065
< 65	Reference			Reference		
≥ 65	1.324	1.018–1.721		1.285	0.985–1.676	
Gender			0.966			
Female	Reference					
Male	1.006	0.759–1.333				
Postoperative morbidity			0.321			
No	Reference					
Yes	0.785	0.432–1.325				
BMI, kg/m^2^			0.070			0.064
< 25	Reference			Reference		
≥ 25	0.686	0.456–1.032		0.670	0.438–1.024	
ASA score			0.912			
0–1	Reference					
≥ 2	1.056	0.382–2.912				
Histology type			0.146			
Well/moderately	Reference					
Poorly/undifferentiated	1.378	0.890–2.140				
Tumor size, cm			0.049			0.917
< 4	Reference			Reference		
≥ 4	1.300	1.002–1.688		0.986	0.752–1.292	
Pathologic T stage			0.001			0.011
T2	Reference					
T3-T4a	2.863	1.538–5.327		3.316	1.319–8.342	
Pathologic N stage			0.028			0.042
N negative	Reference					
N positive	2.350	1.095–5.042		2.458	1.338–5.922	
Pathologic TNM stage			0.000			0.012
IB	Reference			Reference		
IIA-IIIC	3.709	1.945–7.076		3.804	1.420–15.249	
Surgical procedure			0.521			
RADG	Reference					
LADG	0.918	0.707–1.192				

*RADG* robotic-assisted distal gastrectomy, *LADG* laparoscopic-assisted distal gastrectomy, *BMI* body mass index, *HR* hazard ratio, *ASA* American Society of Anesthesiologists, *CI* confidence interval

**Table 6:** Univariate and multivariate analyses of the risk factors for disease-free survival in the PSM cohort

Variables	Univariate		*P*	Multivariate		*P*
	HR	95% *CI*		HR	95% *CI*	
Age, years			0.059			0.128
< 65	Reference			Reference		
≥ 65	1.278	0.990–1.650		1.223	0.944–1.586	
Gender			0.852			
Female	Reference					
Male	0.974	0.740–1.282				
Postoperative morbidity			0.267			
No	Reference					
Yes	0.734	0.419–1.275				
BMI, kg/m^2^			0.040			0.059
≥ 25	Reference			Reference		
< 25	1.520	1.018–2.267		1.591	1.049–2.414	
ASA score			0.435			
0–1	Reference					
≥ 2	1.056	0.382–2.912				
Histology type			0.121			
Well/moderately	Reference					
Poorly/undifferentiated	1.415	1.078–1.862				
Tumor size, cm			0.043			0.751
< 4	Reference			Reference		
≥ 4	1.299	1.009–1.674		0.958	0.737–1.246	
Pathologic T stage			0.000			0.019
T2	Reference					
T3-T4a	3.332	1.805–6.149		3.429	1.229–9.568	
Pathologic N stage			0.000			0.032
N negative	Reference					
N positive	2.985	2.080–4.283		2.370	1.075–5.225	
Pathologic TNM stage			0.000			0.024
IB	Reference			Reference		
IIA-IIIC	4.016	2.113–7.635		3.812	1.445–14.325	
Surgical procedure			0.710			
RADG	Reference					
LADG	0.953	0.741–1.227				

*RADG* robotic-assisted distal gastrectomy, *LADG* laparoscopic-assisted distal gastrectomy, *BMI* body mass index, *HR* hazard ratio, *ASA* American Society of Anesthesiologists, *CI* confidence interval

## Discussion

LG has been identified as a safe and feasible alternative technique for AGC treatment for experienced surgeons [4, 7, 8, 28, 29]. Moreover, a previous study of 3552 patients from seven high-volume GC centers in China has reported that RG brings about less operative blood loss, low incidence of overall complications, and comparable oncological results compared with LG [18]. To date, the potential clinical benefits of RG have not been fully confirmed in GC surgery, especially AGC surgery. On this basis, we performed this study to assess the surgical and oncological outcomes of RADG and LADG for AGC. We found that RADG presented less operative blood loss, as well as more retrieved LNs in total and supra-pancreatic areas but a greater operative time and cost compared with LADG. The oncological outcomes of RADG were similar to those of LADG. To the best of our knowledge, the present paper, for the first time, reported a large-scale case study with a comparison between RADG and LADG with D2 LN dissection for AGC; PSM was used to minimize the effect of imbalanced clinicopathological parameters between the two groups.

According to current evidence, longer operative time is often considered a disadvantage of RG [30], limiting the prevalence of RG technology in general surgery. The present study investigated that the mean operation time in RADG group was significantly longer than that in LADG group, consistent with previous multicenter studies [18, 31]. This could be because the preparation and docking time of robots accounted for a larger proportion of the prolonged time during robotic surgery. Moreover, the mean operation time was shorter in both groups than that reported before because our study mainly focused on patients undergoing distal gastrectomy, while the previous study included a higher proportion of patients receiving total or proximal gastrectomy [12, 18].

Nishi’s study, which recruited 451 patients undergoing minimally invasive surgery, showed that RG caused significantly less operative blood loss than LG [15]. In the current study, the operative blood loss was less in RADG group than in LADG group. This finding could be attributed to the advanced robotic surgery systems, including an internal EndoWrist with seven degrees of freedom, three-dimensional vision, tremor filter, and short learning curve, which prevented injuries from the blood vessels and allowed precise dissection and high flexibility for surgeons [32]. Previous studies proved that one critical barrier to the wide RG application in clinical practice was high costs [12, 13, 16, 17, 33, 34]. Our study also found that RADG group had higher medical cost than the LADG group. However, with the advancement of new technologies, we believe that additional costs of robotic surgery will decrease in the future.

The advantages of RG lie in the articulating function of robotic surgery systems, which helps surgeons perform lymphadenectomy around deep-seated vessels more easily, particularly along the splenic artery. However, whether RG could retrieve more LNs still remains controversial [35]. Generally, sufficient retrieved LNs can improve the accuracy of staging [36]. Our study showed that the number of harvested LNs of RADG was significantly more than that of LADG, consistent with the outcomes of a multicenter randomized controlled trial (RCT) [33] and a single-center RCT [34]. In contrast, a meta-analysis including 7275 patients with AGC reported that the number of retrieved LNs did not differ between RG and LG groups [37]. Furthermore, Obama et al. conducted a study including 837 patients with GC undergoing mini-invasive gastrectomy and showed no significant difference in the number of retrieved LNs between robotic and laparoscopic groups [20]. These differences might result from different types of stomach resection, surgical skills, and experience of surgeons. In the current study, to reduce the differences in stomach resection types among patients, only patients accepting distal gastrectomy were enrolled. Furthermore, all cases of distal gastrectomy were performed by extensively experienced teams who overcame the learning curve of minimally invasive surgery.

D2 LN dissection is a technically demanding procedure in radical gastrectomy for AGC, especially in high BMI patients [14]. However, we found that RADG had more advantages for patients with high BMI than those with normal BMI, as can be seen in the lower severe postoperative complication rate and the approximate operation time to that of LADG. The underlying cause for this discrepancy is that stable exposure and use of a wristed instrument with robotic surgery systems may help to efficiently perform this complex procedure. Thus, we could conclude that the technical advantages of the robotic system might be more obvious in complex surgical fields caused by excessive intra-abdominal fat and thick abdominal walls.

Although several studies have reported the postoperative complications, the results are still elusive [16, 38–40]. A previous study demonstrated that RG showed similar postoperative morbidity to that of LG [16]. A recent meta-analysis also revealed no significant differences in the overall or severe complication rates between robotic and laparoscopic groups [30]. However, a single-center RCT demonstrated a lower postoperative complication rate in RDG group than in LDG group [34]. In addition, a retrospective multicenter study covering 3552 patients with GC showed that the morbidity rates were 12.6% in RG group and 15.2% in LG group, with a significant difference [18]. Moreover, previous single-center studies [14, 15, 27, 40] and a meta-analysis [41] have reported that the RG complication rate varied from 5.2 to 24.1%. In our study, the postoperative complication rate of RADG group was comparable to that of LADG group (13.7% vs. 16.6%, *P* = 0.242). Furthermore, no significant difference was shown in the severe complication rate between two groups (4.9% vs. 6.3%, *P* = 0.363). These differences might be attributed to multiple reasons. For example, previous studies [29, 31, 32, 35] mainly focused on patients with early GC, who presented lower morbidity and mortality than those with AGC. In response to this, further RCTs of robotic distal gastrectomy with D2 lymphadenectomy should be performed.

The oncological outcomes in RG were identified to be similar to those in LG in GC treatment [12, 15, 18–20, 40, 42, 43]. However, most studies were limited by short follow-up time and small sample size. More importantly, most patients in these studies were in an early stage of GC [12, 13, 42]. Therefore, whether the technical superiority of the robotic system in lymphadenectomy could improve long-term survival outcomes still remains unclear. In our study, no significant differences were observed between the two groups in the 3-year OS rate and DFS rate with a median follow-up of 39 months. Furthermore, multivariate analysis indicated that the surgical approach was not an independent prognostic factor for DFS and OS. Such outcomes indicated that not surgery type but tumor biology is the greater determinant of the long-term survival of patients. Therefore, we concluded that RADG and LADG had similar long-term survival outcomes for AGC. However, in the future, large and multicenter RCTs are expected to assess the reliability of these results.

Our study has several limitations. First, some selection bias existed owing to the retrospective nature of the analyses. Second, although we performed a subgroup analysis according to BMI, the study on different related factors was not enough. Third, the proportion of high BMI patients in this study was relatively small, and the results may be less pertinent to Western populations, where high BMI patient is more common. Further research especially large prospective randomized studies are necessary. Fourth,

patients receiving neoadjuvant chemotherapy or radiation were excluded from the present study. Therefore, the superiority of RADG after neoadjuvant chemotherapy or radiation remained unclear. Fifth,

the adjuvant treatments may influence patient survival, potentially limiting the generalizability of these findings. Finally, all patients undergoing curative mini-invasive distal gastrectomy were treated by experienced surgeons, which might limit the generalizability of the study results.

## Conclusion

In conclusion, RADG appears to be a safe and feasible procedure and could serve as an alternative treatment for AGC in experienced centers. In addition, the benefits of RADG might be more evident in high BMI patients. However, RADG did not significantly improve oncological outcomes. In the future, large and multicenter RCTs are needed to validate the reliability of our results.

## Data Availability

The datasets used in this study are available from the corresponding author on reasonable request.

## Abbreviations

RADG: Robotic-assisted distal gastrectomy
LADG: Laparoscopic-assisted distal gastrectomy
PSM: Propensity score matching
BMI: Body mass index
ASA: American Society of Anesthesiologists
TNM: Tumor-node-metastasis
GC: Gastric cancer
AGC: Advanced gastric cancer
LNs: Lymph nodes
RG: Robotic gastrectomy
LG: Laparoscopic gastrectomy
OS: Overall survival
DFS: Disease-free survival
RCT: Randomized controlled trial
